# Saliva Samples as A Tool to Study the Effect of Meal Timing on Metabolic And Inflammatory Biomarkers

**DOI:** 10.3390/nu12020340

**Published:** 2020-01-28

**Authors:** Katharina Kessler, Silke Hornemann, Natalia Rudovich, Daniela Weber, Tilman Grune, Achim Kramer, Andreas F. H. Pfeiffer, Olga Pivovarova-Ramich

**Affiliations:** 1 Department of Clinical Nutrition, German Institute of Human Nutrition Potsdam-Rehbruecke, 14558 Nuthetal, Germany; kk639@cam.ac.uk (K.K.); silkehornemann@o2mail.de (S.H.); natalia.rudovich@spitalbuelach.ch (N.R.); Andreas.Pfeiffer@charite.de (A.F.H.P.); 2 German Center for Diabetes Research (DZD), 85764 München-Neuherberg, Germany; scientific.director@dife.de; 3 Department of Endocrinology, Diabetes and Nutrition, Campus Benjamin Franklin, Charité University of Medicine, 12203 Berlin, Germany; 4 Biomineral Research Group, Department of Veterinary Medicine, University of Cambridge, Cambridge CB3 0ES, UK; 5 Division of Endocrinology and Diabetes, Department of Internal Medicine, Spital Bülach, 8180 Bülach, Switzerland; 6 Department of Molecular Toxicology, German Institute of Human Nutrition Potsdam-Rehbruecke (DIfE), 14558 Nuthetal, Germany; Daniela.Weber@dife.de; 7 NutriAct-Competence Cluster Nutrition Research Berlin-Potsdam, 14558 Nuthetal, Germany; 8 German Center for Cardiovascular Research (DZHK), 10785 Berlin, Germany; 9 Institute of Nutrition, University of Potsdam, 14558 Nuthetal, Germany; 10 Laboratory of Chronobiology, Institute for Medical Immunology, Charité University of Medicine, 10117 Berlin, Germany; achim.kramer@charite.de; 11 Reseach Group Molecular Nutritional Medicine, Dept. of Molecular Toxicology, German Institute of Human Nutrition Potsdam-Rehbruecke, 14558 Nuthetal, Germany

**Keywords:** meal timing, saliva, circadian clock, adiponectin, resistin, visfatin, insulin, melatonin, cortisol, cytokines

## Abstract

Meal timing affects metabolic regulation in humans. Most studies use blood samples for their investigations. Saliva, although easily available and non-invasive, seems to be rarely used for chrononutritional studies. In this pilot study, we tested if saliva samples could be used to study the effect of timing of carbohydrate and fat intake on metabolic rhythms. In this cross-over trial, 29 nonobese men were randomized to two isocaloric 4-week diets: (1) carbohydrate-rich meals until 13:30 and high-fat meals between 16:30 and 22:00 or (2) the inverse order of meals. Stimulated saliva samples were collected every 4 h for 24 h at the end of each intervention, and levels of hormones and inflammatory biomarkers were assessed in saliva and blood. Cortisol, melatonin, resistin, adiponectin, interleukin-6 and MCP-1 demonstrated distinct diurnal variations, mirroring daytime reports in blood and showing significant correlations with blood levels. The rhythm patterns were similar for both diets, indicating that timing of carbohydrate and fat intake has a minimal effect on metabolic and inflammatory biomarkers in saliva. Our study revealed that saliva is a promising tool for the non-invasive assessment of metabolic rhythms in chrononutritional studies, but standardisation of sample collection is needed in out-of-lab studies.

## 1. Introduction

It is becoming increasingly evident that timing of food intake is an important factor influencing energy and metabolic homeostasis and the risk of obesity [1]. Rodent studies have repeatedly shown how timing of food intake can shift metabolic outcomes: mice fed a high-fat diet during the light phase, i.e., the rest phase in these nocturnal animals, gain more body weight relative to littermates fed during the dark phase, i.e., active phase [2], whereas rodents with access to food restricted to the dark phase seem protected against obesity, glucose intolerance, leptin resistance, and other metabolic disturbances [3,4]. In humans, the time at which the main meal is consumed influences the risk of obesity and, at least for overweight and obese people, eating most calories early in the day has been suggested to be beneficial for weight management and metabolism [5]. For example, it has been reported that obese individuals who ate their lunch, which is the main meal for this studied population, after 15:00 lost less weight on a hypocaloric Mediterranean diet than individuals who consumed their lunch before 15:00 [6]. A similar study in overweight and obese individuals indicated that individuals who consume more energy at dinner, compared to breakfast, lose less weight and have higher overall daily glucose, insulin, ghrelin, and hunger scores [7]. In normal weight individuals, a range of studies have suggested that late and delayed eating is associated with reduced energy expenditure / substrate oxidation and a general deterioration in metabolic function, whilst showing no clear results regarding weight gain [5]. 

Recently, it has been suggested that certain time windows are more suitable than others for the consumption of certain macronutrients. For instance, Bray et al. showed in mice that consumption of a high-fat diet at the end of the active phase leads to an increased body weight, glucose intolerance, hyperinsulinemia, hypertriglyceridemia and hyperleptinemia as opposed to the consumption of the same high-fat diet at the beginning of the active phase [8]. Epidemiological studies in humans show that increasing carbohydrate intake at breakfast while simultaneously reducing fat intake seems protective against the development of diabetes and metabolic syndrome [9,10]. Similarly, we recently showed that a diet in which fat is mainly eaten in the morning and carbohydrates mainly in the evening (compared with the reverse order) worsens glycaemic control in people with prediabetes [11] and alters substrate oxidation and adipokine secretion [12]. These studies indicate that timing of carbohydrates and fat in an important factor influencing metabolic health. 

Being rapidly and easily available and non-invasive, saliva has taken an increased attractivity in disciplines such as medicine, dentistry, pharmacotherapy and epidemiology [13]. Saliva refers to the clear, slightly acidic, hypotonic and mucoserous exocrine biological fluid synthesised and secreted by the salivary glands [14]. The major salivary glands (parotid, submandibular, sublingual) produce about 90% of the total saliva, while the minor glands, although being numerous in numbers (300–1000 units), produce only about 10%. Whole mouth saliva, as opposed to saliva produced by specific glands, is a mixture of oral fluids rich in water (~99%), inorganic salts, enzymes, polypeptides and proteins [13,14]. In addition, blood-based molecules enter the highly vascularized salivary glands, and as such, alterations in the composition of blood may be mirrored by modifications in the biochemical composition of saliva [13]. In particular, stimulated whole mouth saliva has been proposed to resemble plasma in its composition [15]. Although saliva sampling can be challenging and suitability of sampling methods needs critical reviewing [14], it is perhaps not surprising that numerous studies seek to use saliva samples in human health monitoring. For example, saliva samples have been used as monitoring tool of therapy progression [16,17], in psoriasis [14], obesity and type 2 diabetes [18,19]. 

Circadian studies have recently revealed that ~15% of all identified metabolites in saliva are under circadian control [20]. Nevertheless, the vast majority of studies use plasma and serum samples for their analysis, and only a few have used saliva samples to investigate circadian rhythms or effects of meal timing. For example, Garaulet’s group recently compared diurnal rhythms in salivary microbiota upon early and late eating conditions [21]. Here, we tested if stimulated whole mouth saliva samples, collected every four hours over 24 hours, could be used to study the effect of timing of carbohydrate and fat intake on diurnal rhythms of metabolic and inflammatory biomarkers.

## 2. Materials and Methods

### 2.1. Study Design 

29 non-obese men without diabetes and without shift work completed this randomized controlled, cross-over trial. The study protocol and informed consent document were approved by the Medical Ethics Committee of Charité University Medicine, Berlin, Germany (EA2/074/12), and were in accordance with the Helsinki Declaration of 1975. All subjects gave written informed consent. The study was registered at clinicaltrials.gov as NCT02487576. Details of the study design, the recruitment of participants, inclusion and exclusion criteria, and dietary interventions were published elsewhere [11,12]. 

**Figure 1:**
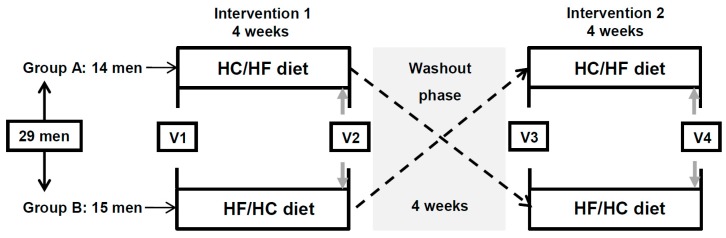
Study design. 29 non-obese men participated in this cross-over trial and were randomized to two 4-week isocaloric dietary interventions, which were separated by a 4-week washout phase. Before (V1 and V3) and after (V2 and V4) each dietary intervention, participants were clinically examined. Prior to V2 and V4 (i.e., last day of the intervention, indicated by grey arrows), saliva samples were collected every 4 hours over 24 hours. HC/HF diet, isocaloric high-carb meals until 13:30 and isocaloric high-fat meals between 16:30 and 22:00; HF/HC diet, reversed order of meal sequence; V, visit.

In brief, in this cross-over trial, participants underwent two 4-week isocaloric dietary interventions, which were separated by a 4-week washout phase (Figure 1): in the HC/HF phase, participants consumed a high-carb diet (breakfast and lunch) until 13:30 and a high-fat diet (snack and dinner) between 16:30 and 22:00; in the HF/HC phase, a high-fat diet was consumed until 13:30 and a high-carb diet between 16:30 and 22.00. The macronutrient composition of the two phases is as follows: HC/HF - 65 energy percent (EN%) carbohydrates (CHO), 20 EN% fat and 15 EN% protein; HF/HC - 35 EN% CHO, 50 EN% fat and 15 EN% protein. As the calories were evenly distributed between the morning (until 13:30) and evening (16:30 to 22:00) block, the resulting daily macronutrient composition was 50 EN% CHO, 35 EN% fat (14 EN% saturated fatty acids) and 15 EN% protein in both diets. For each participant, individual isocaloric dietary plans were designed. These plans met the target macronutrient composition of both diets and considered individual food preferences which were obtained from food records handed out prior to trial commencement [11,12]. Participants were asked to daily document their food selection, consumed amount and time of each meal (Figure S1). Analysis of dietary protocols showed a good compliance to both diets [11]. 

Before (Visit 1 and Visit 3) and after (Visit 2 and Visit 4) each interventions, participants were examined clinically as previously published [11,12]. After each intervention period (Visit 2 and Visit 4), participants underwent two meal tolerance tests (MTT): the first MTT started at 09:00, the second at 15:40 (Figure 1). As per the participant’s previous intervention, the test meals were either high in carbohydrates (MTT-HC; 835 kcal; 64.8 EN% CHO, 14.8 EN% protein, 20.3 EN% fat) or high in fat (MTT-HF; 849 kcal; 35.3 EN% CHO, 15.1 EN% protein, 49.6 EN% fat). Blood samples were drawn from the forearm vein four times during the daytime (at 8:35, 12:15, 15:35 and 18:55, i.e., before and 180 min after completion of each test meal), because 24-hour blood sample collection was technically not feasible. Blood samples for insulin assessment were taken before and 30, 60, 90, 120 and 180 min after completion of each test meal.

### 2.2. Saliva Sample Collection

At the last day of each intervention period (i.e., day before Visit 2 and Visit 4, Figure 1), samples of stimulated whole mouth saliva, which has been reported to resemble plasma in its composition [15,16], were collected, at home, every 4 h throughout a 24 h day, using a saliva cotton roll commercial collection device as per manufacturer’s instructions (Salivettes®, Sarstedt, Germany). In brief, participants were asked to remove the cotton swap from the Salivette®, place it into their mouth and chew it for about 60–120 sec to stimulate salivation. To avoid any contamination, participants were asked to refrain from using their hands when returning the swap with the absorbed saliva to the Salivette®; instead, they were encouraged to slide the swap into the Salivette® using their mouth. Times of collection were at 04:00, 08:00, 12:00, 16:00, 20:00 and 24:00. Participants were thoroughly instructed to ensure high quality samples. Participants were instructed to refrain from tooth brushing and eating for at least 30 min prior to collecting samples. Where this collided with their usual meal times (Figure S1), participants were asked to pre- and postpone their meals (within the allowed allocated time frames, i.e., until 13:30 for breakfast and lunch and 16:30 to 22:00 for a snack and dinner). Participants were also asked to follow their habitual sleep and wake times during the day of collection. For time points at which participants were usually asleep, they were told to keep exposure to artificial light to a minimum. In addition, participants were asked to keep samples at 4 °C and cooling packs were provided to ensure samples were kept cool whilst being transported to the lab, where samples were processed. 

### 2.3. Sample Analysis 

At arrival, salivettes were centrifuged at 10,000 g for 10 min at 4 °C, and supernatants were stored at -80 °C until analysis. Salivary protein concentrations were measured by Bradford method. Salivary pH levels were assessed using Schott pH meter CG840 and InLab minielectrode (Mettler Toledo).

For salivary cortisol and melatonin, commercially available ELISA kits (RE52611 and RE54041, IBL, Hamburg, Germany) were used according to manufacturer´s instructions. Multiplex magnetic bead panels on a Luminex 200 platform (BioRad, Germany) were used for measurement of all other salivary markers, according to manufacturers´ instructions. For the measurement of salivary adiponectin and resistin, a 2-plex assay was used (HADCYMAG-61K, Millipore, USA); samples were 1:2 diluted. A 5-plex assay (BioRad, USA) was used for determination of salivary IL-6 (171-B5006M), MCP-1 (171-B5021M) and insulin, visfatin and leptin (all 17001408, Bio-Plex Pro Human Diabetes). A 4-plex assay (HMHEMAG-34K-04, Millipore, USA) was used to determine salivary ghrelin, peptide YY, insulin and glucagon. Salivary ghrelin and glucagon were also measured with the Bio-Plex Pro Human Diabetes (17001408).

Blood samples were processed as follows: S-Monovette® (Sarstedt, Germany) containing EDTA, lithium-heparin or citrate were used for plasma, depending on the further analysis, and were centrifuged (10 min, 3,000 × rpm, 4 °C) immediately after sampling, whereas samples for serum were allowed to clot 10 min prior to centrifugation. Samples were stored at -80 °C until analysis. Routine laboratory markers in plasma were measured using standard methods (ABX Pentra 400; HORIBA, ABX SAS, Grabels, France). Commercial ELISA were used for measurement of insulin (Mercodia, Uppsala, Sweden), adiponectin (BioVendor, Kassel, Germany), interleukin 6 (IL-6), monocyte chemoattractant protein-1 (MCP-1) (all from BioTechne GmbH, Wiesbaden, Germany), visfatin/NAMPT (Biomol, Hamburg, Germany) and cortisol (IBL International, Hamburg, Germany) in serum.

### 2.4. Statistical Analysis

Statistical analyses were performed with SPSS v.20 (SPSS, Chicago, IL, USA). Repeated measures two-way ANOVA was performed to determine effects of diet, time and diet*time interaction. Depending on sample distribution, Student’s t-test or Wilcoxon test were used to determine difference between two groups. Daytime levels of biomarkers were calculated as the average level from four time points of data collection (i.e., at 08:00, 12:00, 16:00, and 20:00 for saliva samples and at 8.35, 12.15, 15.35 and 18.55 for blood samples). Correlations of daytime levels in saliva and blood were calculated using Pearson or Spearman tests, depending on sample distribution. P values < 0.05 were considered significant in all analyses. All data are presented as means ± standard error of the mean (SEM). 

## 3. Results

### 3.1. Study Population and Adherence to Dietary Interventions

32 generally healthy men were enrolled in this randomized controlled, cross-over trial, and 29 men (age 45.9 ± 2.5 years, BMI 27.1 ± 0.8 kg/m^2^, 18 subjects with normal glucose tolerance and 11 subjects with impaired fasting glucose/impaired glucose tolerance) completed both dietary interventions. Participants had no history of shiftwork and followed a regular life style. Adherence to dietary plans was good, with similar compliances for both diets. There was no statistical difference in energy intake (*p* = 0.540), macronutrient composition (carbohydrates: *p* = 0.627; protein: *p* = 0.922; fat: *p* = 0.705), amount of saturated fatty acids (*p* = 0.115), fiber (*p* = 0.064) and starch (*p* = 0.086) or glycemic index (*p* = 0.461) between the two diets (Table S1). Body weight was nearly stable with no differences between the two diets, and fasting glucose decreased after both diets (Table 1). 

### 3.2. Detectable Salivary Markers

The hormones cortisol, melatonin, insulin, visfatin, adiponectin and resistin and the inflammatory markers IL-6 and MCP-1 were above lower limit of quantification in the saliva samples. Ghrelin, peptide YY, glucagon and leptin were only marginally or not at all detectable. 

### 3.3. Diurnal Rhythms of Salivary Markers

**Figure 2:**
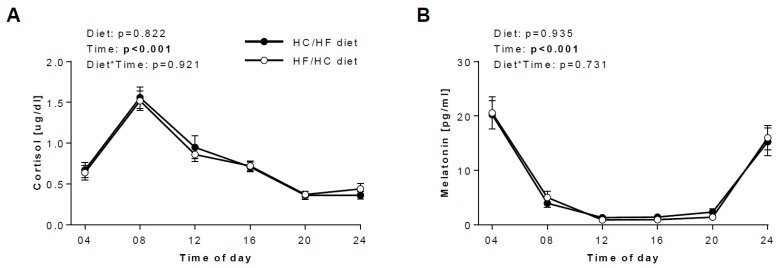

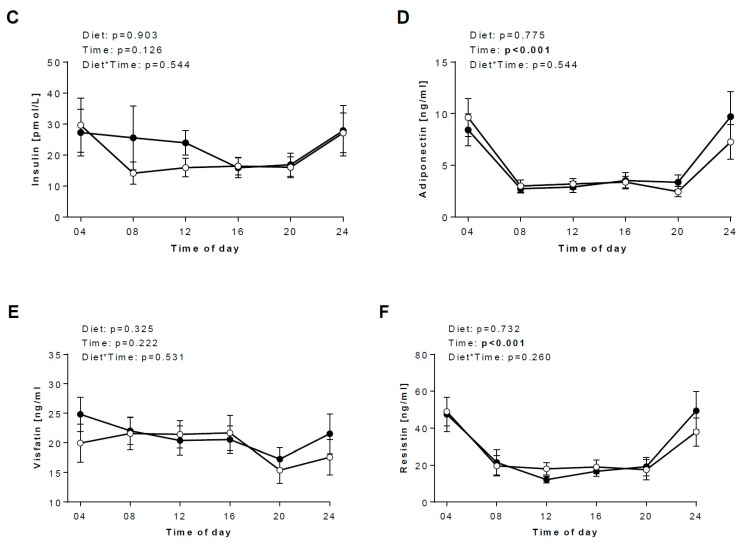
Salivary metabolic biomarkers in response to the HC/HF and HF/HC diet. Effects of the HC/HF diet (black circles) and the HF/HC diet (open circles) on 24-h concentrations of (**A**) cortisol (*n* = 24), (**B**) melatonin (*n* = 20), (**C**) insulin (*n* = 15), (**D**) adiponectin (*n* = 20), (**E**) visfatin (*n* = 22) and (**F**) resistin (*n* = 23). Repeated measures two-way ANOVA was applied to determine the effect of diet, time and diet*time interaction. Data are means ± SEM. HC/HF diet, isocaloric high-carb meals until 13:30 and isocaloric high-fat meals between 16:30 and 22:00; HF/HC diet, reversed order of meal sequence.

We investigated whether detectable salivary biomarkers show diurnal variations and whether these are similar to the daytime patterns in blood serum which were available for the most of studied biomarkers (cortisol, insulin, visfatin, adiponectin, IL-6, and MCP-1)**.** Salivary levels of cortisol and insulin were 10 times lower, and adiponectin 1000 times lower compared with blood levels (Figure 2, Figure S2). Visfatin in saliva was 10 times higher than in serum, whereas IL-6 and MCP-1 showed similar levels in saliva and serum (Figure 2, Figure S2).

Salivary cortisol and melatonin concentrations show a profound diurnal variation, with peak levels at 08:00 for cortisol and nadir levels at 12:00 for melatonin (Figure 2A,B). Daytime pattern of salivary cortisol was very similar to the blood (Figure S2A), and levels in saliva and blood correlated significantly (*r* = 0.308, *p* = 0.019).

Salivary insulin levels showed a tendency to be higher upon the high-carb food in the morning, but high interindividual variation was found (Figure 2C). Although blood pattern demonstrated more pronounced variation (Figure S2B), mean daytime levels in saliva and blood positively correlated (*r* = 0.277, *p* = 0.035). 

Salivary adiponectin and resistin further display diurnal oscillations (Figure 2D and 2F), with peak concentrations occurring at night time and nadir levels around midday. Salivary visfatin showed no diurnal variation (Figure 2E), and its mean daytime levels correlated with blood values (*r* = 0.301, *p* = 0.022) (Figure S2D). 

**Figure 3:**
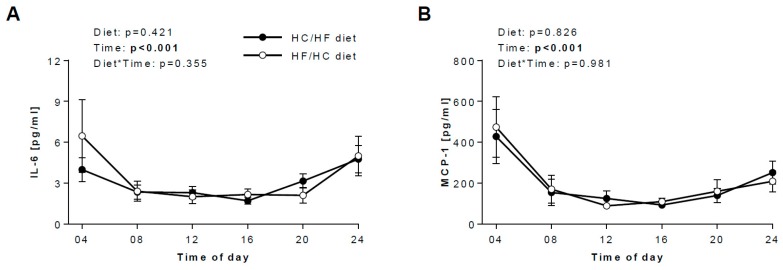
Salivary inflammatory biomarkers in response to the HC/HF and HF/HC diet. Effects of the HC/HF diet (black circles) and the HF/HC diet (open circles) on 24-h concentrations of (**A**) IL-6 (*n* = 14) and (**B**) MCP-1 (*n* = 22). Repeated measures two-way ANOVA was applied to determine the effect of diet, time and diet*time interaction. Data are means ± SEM. HC/HF diet, isocaloric high-carb meals until 13:30 and isocaloric high-fat meals between 16:30 and 22:00; HF/HC diet, reversed order of meal sequence, IL-6, interleukin 6; MCP-1, monocyte chemoattractant protein-1.

IL-6 and MCP-1 in saliva demonstrate a similar pattern as adiponectin and resistin (Figure 3): both markers peak around 04.00 and after reaching the nadir around midday, the concentrations of both markers progressively rise for the rest of the day. Again, salivary pattern of IL-6 and MCP-1 mirrored blood profiles (Figure S2E,F), although no significant correlations were found.

We then assessed the salivary protein concentrations and pH levels to test whether they might affect the detected biomarker concentrations. We found that salivary protein concentrations were increased and pH values decreased at night (Figure S3).

### 3.4. Daily hormonal profiles in response to the diets 

No effects of diet or time*diet interaction of the patterns of any salivary biomarkers were found (Figure 2, Figure 3), suggesting that the time at which mainly carbohydrates and fat are being consumed has only a small or even negligible effect on salivary concentrations of metabolic and inflammatory biomarkers. 

## 4. Discussion

In this study, we investigated how the intake of carbohydrate and fat at different times of the day affects salivary biomarkers in humans. The main findings of this study are: (1) saliva is a promising tool for determination of certain metabolic and inflammatory markers; (2) diurnal variations of metabolic markers are pronounced and distinct in saliva; (3) in saliva, secretion pattern of metabolic and inflammatory markers do not depend on the timing of carbohydrate and fat intake.

In recent years, saliva has gained importance in the study of metabolic diseases, including obesity and type 2 diabetes [18,19,22,23]. Different components of blood have been demonstrated to enter saliva, including metabolic and inflammatory markers, and for several it has been shown that their salivary concentrations correlate with their concentrations in blood, including insulin [24,25], ghrelin [26], adiponectin [25,27], leptin [28], resistin [27], CRP [25,29], cortisol [30] and melatonin [31]. Perhaps not surprisingly, saliva has therefore repeatedly been proposed as a promising tool for the study of metabolic diseases. In the circadian field, saliva samples have very recently been used to investigate, e.g., the effect of meal timing on salivary microbiota [21] or the effect of night work on salivary cytokines [32]. In our study, we used salivary samples to study the effect of meal timing (timing of carbohydrate and fat intake) on selected metabolic and inflammatory markers. Using multiplexing array panels, as described before for saliva [23,25], we were able to detect salivary cortisol, melatonin, insulin, visfatin, adiponectin, resistin, IL-6 and MCP-1. Similarly with the literature data, salivary concentrations of cortisol, insulin, and visfatin correlated with their concentrations in blood.

Further, most detected biomarkers demonstrated distinct diurnal variations, mirroring reports in blood. Except for well-known circadian rhythms in salivary melatonin and cortisol [33], we also showed diurnal oscillations of metabolic and inflammatory markers so far poorly studied in human saliva. Components of circadian clocks are known to be tightly involved in the regulation of metabolism and immune response [34]. Many components of lipid homeostasis are under circadian control including intestinal lipid transport, de novo lipid synthesis and adipokine secretion [35]. Similarly, in immune response, immune cell number, functions such as cytokine expression, phagocytosis and lytic activity, and expression of corresponding genes are subjected to circadian control [33,36]. In our study, we show profound circadian regulation of salivary adiponectin, resistin, IL-6 and MCP-1 concentrations. The concentrations of these adipokines and cytokines are increased at late in the evening / night and decreased throughout the day, which mirrors diurnal patterns shown in blood [12]. In serum, IL-6 and MCP-1 concentrations progressively rise in the course of the day, whereas adiponectin shows a downward trend throughout the day [12]. A similar pattern has been described by others for serum adiponectin [37]. Resistin has been reported to exhibit diurnal variation in plasma/serum in rodents [38], while reports in humans seem rare [39]. Taken together, our results indicate that adipokines and inflammatory markers show very similar circadian patterns in both saliva and blood, and suggest that saliva may be an excellent tool for chrononutritional investigations. 

Interestingly, in our study, salivary secretion patterns of metabolic and inflammatory markers do not depend on timing of fat and carbohydrate intake. For adiponectin, IL-6 and MCP-1, this observation is in line with our previous report [12], where we showed that the HC/HF diet, in comparison to the HF/HC diet, did not change their average concentrations and/or diurnal variations. For melatonin and cortisol, it is generally thought that meal timing has only a limited influence on melatonin and cortisol [40], both of which are indicative of the central circadian clock in the suprachiasmatic nucleus, although a very recent trial suggests that early time restricted feeding may have been a direct impact on cortisol, leading to an increased amplitude [41]. Our study suggests that timing of carbohydrate and fat intake also has only limited influence on cortisol and melatonin, i.e., the oscillation patterns in saliva did not differ between our two diets. This is noteworthy as we could previously show that a shift from a carbohydrate-rich diet to a fat-rich diet leads to a delay in the salivary cortisol rhythms [33]. 

Finally, it is well accepted that the glucose metabolism is under circadian control [41]. Both meal composition and meal timing strongly influence blood insulin concentrations [11]. Interaction of rhythms in insulin sensitivity and insulin secretion result in the peak of the glucose tolerance in the biological morning, at least in non-diabetic subjects [1,41]. Interestingly, in our saliva samples, timing of carbohydrate and fat intake has only a minimal influence on insulin concentrations, i.e., there was no difference between the two diets. The difference in results between saliva and serum might be explained by the time of meal intake: in our published reports [11,12], the serum samples were collected during a strictly controlled clinical investigation days, which was undertaken at the outpatient department of the institute. During these clinical investigation days, the meals were identical for all participants and the time of meal intake was highly controlled and the same across all participants. In contrast, the saliva samples were collected at home, i.e., under less controlled conditions. We cannot rule out that the samples, primarily at 08:00, 12:00 and 20:00, were collected under preprandial conditions for some participants and under postprandial conditions for others (Figure S1). Undoubtedly, this may have profound implications, at least for insulin that dramatically increases shortly after the meal intake, particularly if high in carbohydrates, as we showed in the serum samples (Figure S2B). Meal onset at breakfast (Figure S1B) was most varied among our participants, which may explain the large variation in the salivary insulin concentration at 8:00 (Figure 2C) on the HC/HF diet (but not on the HF/HC diet), although we cannot exclude that the relatively small sample size (*n* = 15) may have contributed to this phenomenon. The small sample size (*n* = 14) may also explain the large variation in salivary IL-6 concentration (Figure 3A). For future studies, a standardisation of conditions for sample collection is needed for biomarkers showing the strong postprandial changes, even if this is difficult in out-of-lab studies.

It has to be noted that the salivary flow rate and pH can affect concentration of biomarkers in saliva [13,42,43]. In our study, we were not able to assess the salivary flow rate because study participants collected samples at home. However, we assessed the salivary protein concentrations, which usually negatively correlates with salivary flow rate [23], and salivary pH levels. We found that salivary pH levels declined at night and protein concentrations increased at night, which is in agreement with literature data [44], and we therefore cannot exclude that these factors affect biomarker concentrations detected at night. Therefore, adjustment for the salivary flow rate can be recommended for the biomarker analysis in chrononutritional studies, which represents the next limitation of the out-of-lab studies where the assessment of the salivary flow rate is not feasible.

Taken together, our study suggests that saliva is a promising non-invasive tool for the determination of circadian patterns of metabolic and inflammatory biomarkers in human studies. Our study revealed salivary biomarkers which could be used for the non-invasive assessment of metabolic rhythms in chrononutritional studies.

## Figures and Tables

**Table 1:** Fasting parameters in response to the diets.

	HC/HF diet			HF/HC diet			P^1^	P_corr_^2^
	Pre	Post	Δ%	Pre	Post	Δ%		
Weight [kg]	86.8 ± 2.8	86.4 ± 2.9	−0.5	87.1 ± 2.9	86.6 ± 2.9	−0.6	0.936	
BMI [kg/m²]	27.0 ± 0.7	26.8 ± 0.8	−0.7	27.1 ± 0.8	26.9 ± 0.8	−0.7	0.917	
Total body fat [%]	24.7 ± 1.5	24.2 ± 1.7	−2.0	24.5 ± 1.7	23.3 ± 1.7	−4.9	0.206	
Triglycerides [mmol/L]	1.15 ± 0.13	1.07 ± 0.11	−7.0	1.25 ± 0.16	1.17 ± 0.11	−6.4	0.666	0.666
Total cholesterol [mmol/L]	5.22 ± 0.17	4.78 ± 0.16	−8.4	5.24 ± 0.18	4.88 ± 0.18	−6.9	0.347	0.273
HDL cholesterol [mmol/L]	1.24 ± 0.04	1.08 ± 0.03	−12.9	1.21 ± 0.04	1.08 ± 0.03	−10.7	0.737	0.704
LDL cholesterol [mmol/L]	3.46 ± 0.16	3.21 ± 0.15	−7.2	3.46 ± 0.17	3.27 ± 0.16	−5.5	0.571	0.527
NEFA [mmol/L]	0.50 ± 0.03	0.45 ± 0.02	−10.0	0.49 ± 0.03	0.45 ± 0.03	−8.2	0.238	0.233
Glucose [mmol/L]	5.91 ± 0.12	5.35 ± 0.07	−9.5**	5.84 ± 0.1	5.33 ± 0.08	−8.7**	0.407	0.415
Insulin [pmol/L]	38.46 ± 4.92	33.06 ± 2.58	−14.0	41.22 ± 5.52	33.72 ± 4.02	−18.2	0.524	0.525
HOMA−IR [mmol· mU· l^−2^]	1.67 ± 0.29	1.29 ± 0.12	−22.8	1.70 ± 0.29	1.24 ± 0.16	−27.1	0.537	0.539

Data are shown as mean ± SEM, *n* = 29. *–*p*-value for the difference from baseline, *p < 0.05, **p < 0.01 (paired Student´s t-test or Wilcoxon test). 1–*p*-value for the comparison of changes after HC/HF and HF/HC diets in the linear mixed model. 2–*p*-value for the comparison of changes after HC/HF and HF/HC diets in the linear mixed model after correction for weight change. HC/HF, isocaloric carbohydrate-rich diet until 13:30 and fat-rich diet between 16:60 and 22:00; HDL, high density lipoprotein; HF/HC, isocaloric fat-rich diet until 13:30 and carbohydrate-rich diet between 16:30 and 22:00

## Supplementary Materials

The following are available online at https://www.mdpi.com/2072-6643/12/2/340/s1, Figure S1: Habitual meal onsets during workdays (A) and weekends (B), Figure S2: Serum levels of biomarkers in response to the HC/HF and HF/HC diet, Figure S3: Saliva protein concentrations (A) and pH values (B) in response to the HC/HF and HF/HC diet, Table S1: Compliance during both dietary interventions.

## Abbreviations

BMI	Body mass index
CHO	Carbohydrates
CID	Clinical investigation day
EN%	Energy percent
FA	Fatty acid
FFA	Free fatty acid
HC/HF	Isocaloric carbohydrate-rich diet until 13:30 and fat-rich diet between 16:30 and 22:00
HF/HC	Isocaloric fat-rich diet until 13:30 and carbohydrate-rich diet between 16:30 and 22:00
IL-6	Interleukin-6
Kcal	Kilo calories
MCP-1	Monocyte chemoattractant protein-1;
MTT-HC	High-carb meal tolerance test
MTT-HF	High-fat meal tolerance test
SFA	Saturated fatty acids
