# Supplementary material for: Saliva Samples as A Tool to Study the Effect of Meal Timing on Metabolic And Inflammatory Biomarkers
Source: Nutrients. 2020 Jan 28;12(2):340. doi: 10.3390/nu12020340 (PMC7071228; doi:10.3390/nu12020340)
Supplement: Supplementary file 1 [file nutrients-12-00340-s001.pdf]

Table S1. Compliance during both dietary interventions

	HC/HF diet			HF/HC diet		
	**06.00 – 22.00**	**06.00 – 13.30**	**16.30 – 22.00**	**06.00 – 22.00**	**06.00 – 13.30**	**16.30 – 22.00**
Energy [KJ]	12073.4 ± 442.3	6197.1 ± 261.3§	5876.0 ± 212.0	11826.3 ± 415.1	6215.0 ± 252.9	5712.7 ± 207.5
CHO [EN %]	49.1 ± 0.7	64.8 ± 0.7	32.7 ± 1.2	48.7 ± 0.7	33.5 ± 0.9	65.0 ± 1.2
Fat [EN %]	36.3 ± 0.7	20.7 ± 0.5	52.4 ± 1.2	36.7 ± 0.8	51.4 ± 1.0	20.6 ± 1.0
Protein [EN %]	14.7 ± 0.2	14.4 ± 0.3	14.9 ± 0.3	14.6 ± 0.2	15.1 ± 0.3	14.4 ± 0.3
SFA [% from total fat]	42.8 ± 1.2	45.4 ± 1.1	41.8 ± 1.4	44.5 ± 1.0	43.9 ± 1.1	44.2 ± 1.4
Dietary GI	58.3 ± 0.5	59.4 ± 0.6	56.0 ± 0.9#	58.7 ± 0.5	59.9 ± 0.7	58.3 ± 0.5
Fibre [g]	35.5 ± 1.9	22.5 ± 1.6§	13.0 ± 0.7	32.9 ± 1.2	14.3 ± 0.6	18.6 ± 0.9
Starch [g]	174.3 ± 10.1	116.7 ± 8.5§	57.4 ± 3.3#	177.1 ± 6.5	79.7 ± 3.3	97.5 ± 4.8

Data are shown as mean ± SEM. §, $p < 0.05$, carbohydrate-rich diet in the morning (06.00 – 13.30) *versus* evening (16.30 – 22.00). #,$p < 0.05$, fat-rich diet in the morning *versus* evening). HC/HF, isocaloric carbohydrate-rich diet in the morning and fat-rich diet in the evening; HF/HC, isocaloric fat-rich diet in the morning and carbohydrate-rich diet in the evening; KJ, kilo joule; CHO, carbohydrates; EN %, energy percent; SFA, saturated fatty acids; GI, glycaemic index.

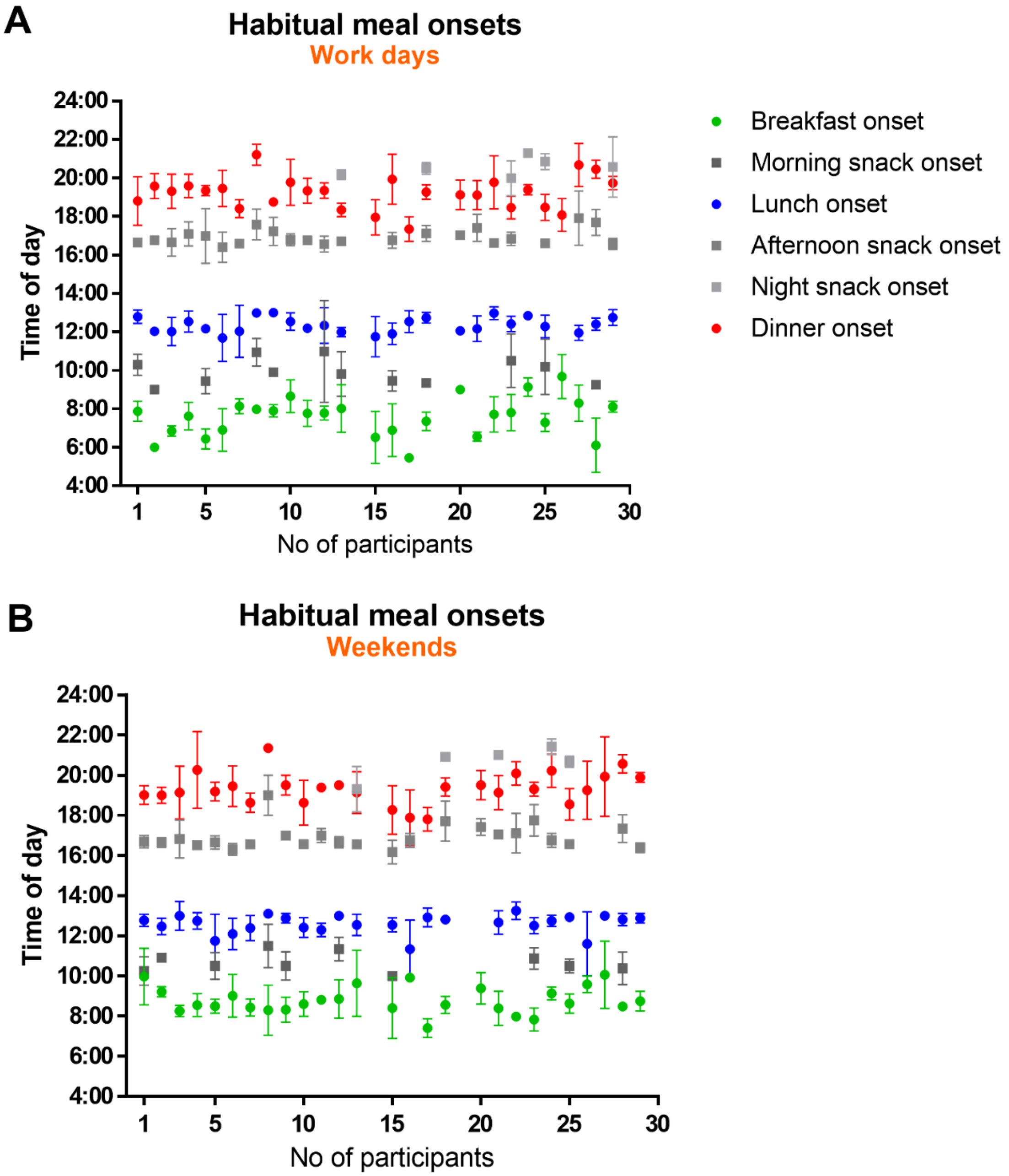

Figure S1. Habitual meal onsets during workdays (A) and weekends (B). Meal times over two weeks were used to calculate average onset times. Meals were included into the graphs if they occurred on at least half of the days analysed (i.e. n≥5 for workdays and n≥2 for weekends). Data are shown as mean ± SD for n=27 participants (two subjects provided insufficient data on their meal times).

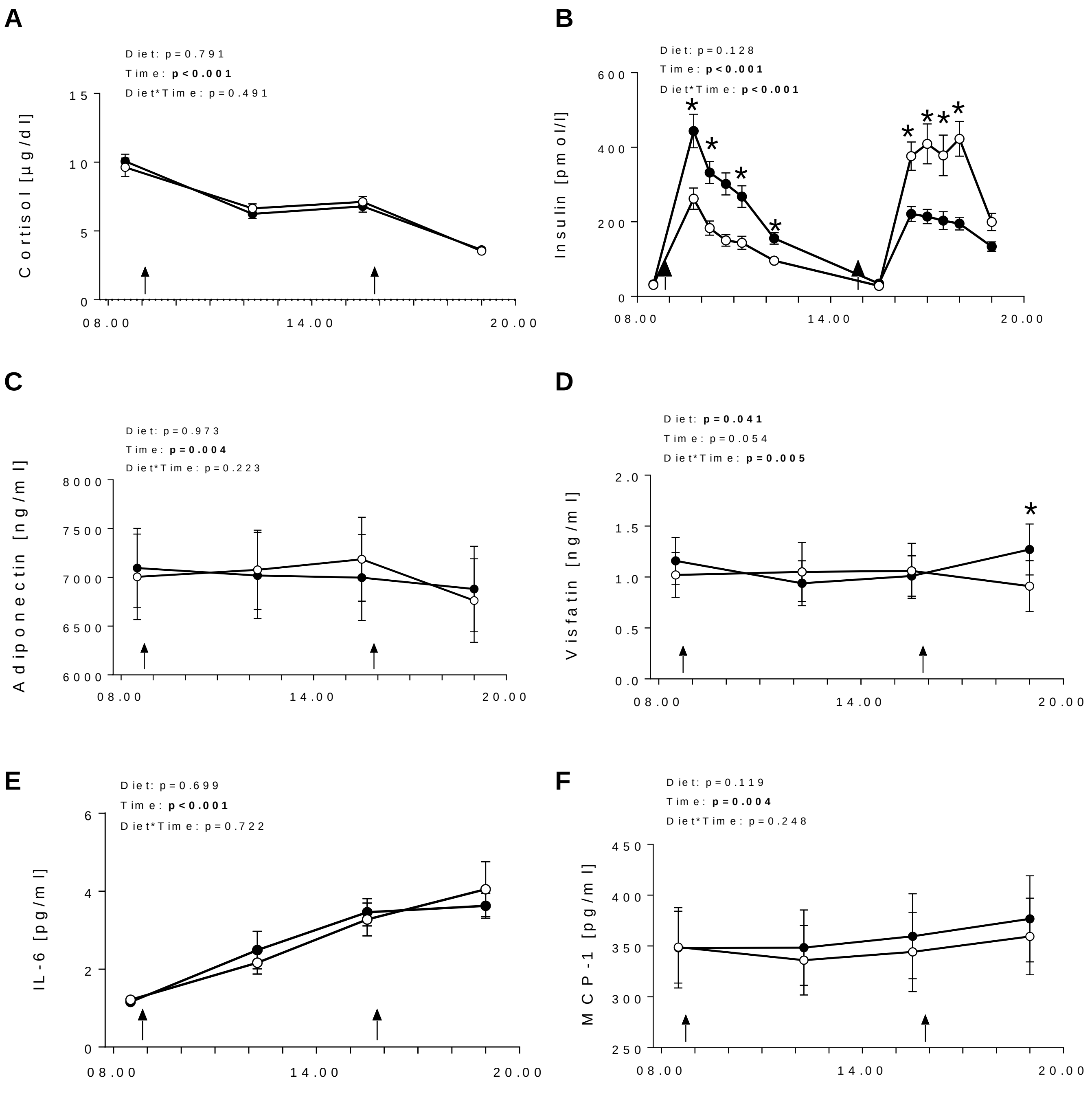

Figure S2. Serum levels of biomarkers in response to the HC/HF and HF/HC diet. Effects of the HC/HF diet (black circles) and the HF/HC diet (open circles) on daytime concentrations of (A) cortisol, (B) insulin, (C) adiponectin, (D) visfatin, (E) IL-6 and (F) MCP-1 (n=29 for all biomarkers). Repeated measures two way ANOVA was applied to determine the effect of diet, time and diet*time interaction. Data are means ± SEM. HC/HF diet, isocaloric high-carb meals until 13:30 and isocaloric high-fat meals between 16:30 and 22:00; HF/HC diet, reversed order of meal sequence. * p>0.05 for HC/HF vs. HF/HC.

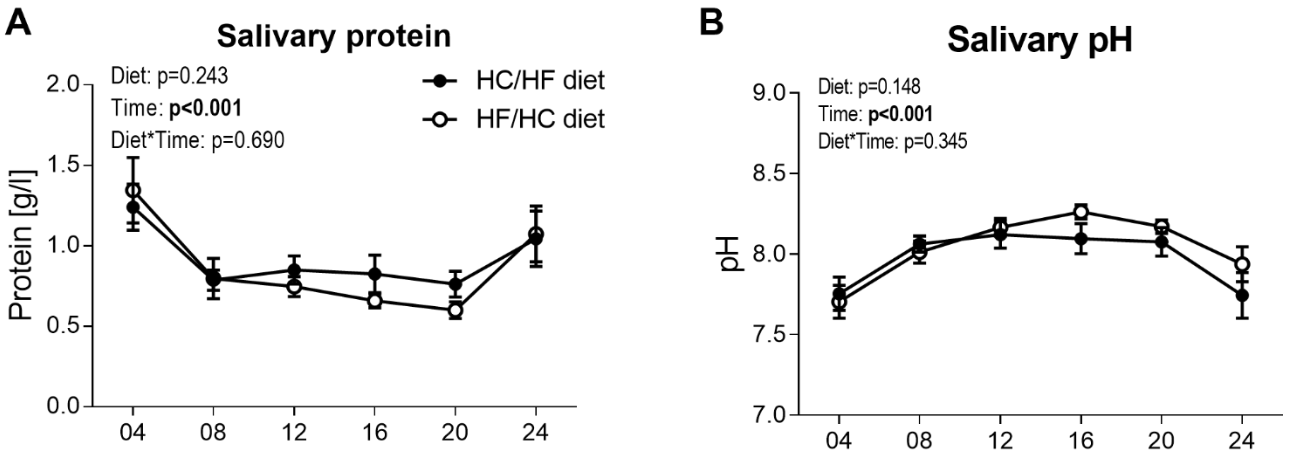

Figure S3. Saliva protein concentrations (A) and pH values (B) in response to the HC/HF and HF/HC diet. (n=29 for all biomarkers). Repeated measures two-way ANOVA was applied to determine the effect of diet, time and diet*time interaction. Data are means ± SEM. HC/HF diet, isocaloric high-carb meals until 13:30 and isocaloric high-fat meals between 16:30 and 22:00; HF/HC diet, reversed order of meal sequence. * p>0.05 for HC/HF vs. HF/HC.
